# Prevalence and Predictors of Current and Former Tobacco Use among Older Adults in Indonesia

**DOI:** 10.31557/APJCP.2019.20.2.395

**Published:** 2019

**Authors:** Supa Pengpid, Karl Peltzer

**Affiliations:** 1 *ASEAN Institute for Health Development, Mahidol University, Salaya, Thailand,*; 2 *Department of Research and Innovation, University of Limpopo, Turfloop,*; 3 *HIV/AIDS/STIs and TB Research Programme, Human Sciences Research Council, Pretoria, South Africa. *

**Keywords:** Tobacco use, health variables, older adults, Indonesia

## Abstract

**Purpose:**

The study aims to describe sociodemographic and health variable indices related to current and former tobacco use among older adults who participated in the Indonesia Family Life Survey (IFLS-5) in 2014-15.

**Materials and Methods:**

A national population-based cross-sectional study was conducted with a probability sample of 8,001 aged 50 years or older Indonesians.

**Results:**

The overall prevalence of current tobacco use was 33.3% (62.2% in men and 6.5% in women) and former tobacco use was 9.8% (17.4% among men and 2.8% in women), of which 64.4% quit tobacco use when 50 years and older. In multinomial regression analysis, sociodemographic factors (being male, lower education, lower economic status, living in Java and rural residence) and health variables (cancer or malignant tumour, depression symptoms, functional disability and inadequate fruit and vegetable consumption) were associated with current tobacco use. In addition, being overweight or obese, having had a stroke, and other lung conditions were inversely associated with current tobacco use. Further, in adjusted analysis, sociodemographic factors (being 70 years and older, being male, living in Sumatra) and having chronic conditions (dyslipidemia, heart problems, asthma, stomach or digestive diseases and functional disability) were associated with former tobacco use.

**Conclusions:**

A high rate of current tobacco use and low rate of former tobacco use was found, particularly among men. The identified risk factors may help to better target this vulnerable population with tobacco cessation programmes.

## Introduction

Some of the highest prevalence of tobacco use are found in low- and middle-income countries, including Indonesia (Sinha et al., 2016; WHO, 2017). In a study among older adults (50 years and older) in six middle-income countries, the prevalence of current tobacco use ranged from 7% in Ghana to 46.5% in India (He et al., 2012). In an older local study in Indonesia, the smoking prevalence among men (65-74 years old) was 84.5% (Ganiwijaya et al., 1995), the current tobacco use rate was 40.7% among 45-64 year-olds and 38.2% in 65 years and older individuals in the Global Adult Tobacco Survey in 2011 in Indonesia (WHO, 2012). In a systematic review in populations 65 years and older, the overall prevalence of tobacco use was 13% (22% male and 8% female) (Marinho et al., 2010). 

Some research seem to suggest that older adults continue tobacco use at a high rate, have less awareness of potential harmful effects and may be more resistant to quit tobacco use (Bratzler et al., 2002; Marinho et al. 2010; Orleans et al., 1994), which emphasises the need to collect information about the tobacco use pattern among older adults and consequently develop adequate tobacco cessation strategies (Chen and Wu, 2015; Marinho et al., 2010). Risk factors for current tobacco use among older adults include sociodemographic and health variables. Sociodemographic risk factors include, younger age (Blazer and Wu, 2012; Kim et al., 2007), no significant decline with older age (Van Heerden et al., 2009), male gender (He et al., 2012; Mini et al., 2014; Peltzer and Phaswana-Mafuya, 2012; Yawson et al., 2013), lower socioeconomic and/or educational status (Mini et al., 2014; Pang et al., 2016), and rural residence in China, Ghana, and India, and urban residence in Mexico (He et al., 2012; Mini et al., 2014). Health variable risk factors for current tobacco use include chronic obstructive pulmonary disease (Nazir and Erbland, 2009), mental health difficulties (Burns et al., 2017), lower life satisfaction (Yawson et al., 2013), poor self-rated health (Bell et al., 2009), insufficient fruits and vegetable consumption (Ko et al., 2011; Peltzer and Phaswana-Mafuya, 2012), and cognitive impairment (Mons et al., 2013). Obesity was negatively associated with tobacco use among older adults in South Africa (Peltzer and Phaswana-Mafuya, 2012).

Similarly, factors associated with tobacco use cessation among older adults include sociodemographic and health variables. Sociodemographic variables include older age (Pang et al., 2016; Yawson et al., 2013), male gender (Peltzer and Phaswana-Mafuya 2012), female gender (Yawson et al., 2013), higher education (Yawson et al., 2013), increasing income levels (Yawson et al., 2013) and among urban residence (Yawson et al., 2013). Health variables associated with tobacco use cessation include higher prevalence of CVDs (Sumartono et al., 2011), transient ischemic attacks (Pang et al., 2016), asthma (Pang et al., 2016), heart trouble or angina (Pang et al., 2016), and mental health difficulties (Burns et al., 2017).

There is a lack of studies that analyse the socio-demographic and health characteristics influencing current and former tobacco use behaviour among older adults in Indonesia.

Therefore, the aim of this study was to assess the prevalence of current and former tobacco use and its associated factors among a national population-based sample of older Indonesians who participated in the Indonesia Family Life Survey (IFLS-5) in 2014-15.

## Materials and Methods

*Sample and procedure*

Data were analysed from the 2014-2015 “Indonesia Family Life Survey (IFLS-5)”.

The IFLS-5 used a multistage stratified sampling design representative of 83% of Indonesia’s population, with a response rate of over 90%; more details (Strauss et al., 2016). In the IFLS-5, 16,204 households and 8001 50 years and older individuals were interviewed with complete tobacco use measurements. The IFLS has been approved by ethics review boards of RAND and University of Gadjah Mada in Indonesia (Strauss et al., 2016). Written informed consent was obtained from all respondents prior to data collection.

*Measures*

Tobacco use was assessed with two questions: 1) “Have you ever chewed tobacco, smoked a pipe, smoked self-enrolled cigarettes, or smoked cigarettes/cigars?” (Yes, No) 2) “Do you still have the habit or have you totally quit?”(Still have, Quit) (Strauss et al., 2016). Responses were grouped into never, quitters and current tobacco users.

Socio-demographic factor questions included age, gender, education (none, elementary, high school or higher education), urban-rural residential status, region (Sumatra, Java and major island groups) and subjective socioeconomic background (poor, medium, rich) (Strauss et al., 2016).

Anthropometric measurements. Body weight and height were assessed with standard measures (Strauss et al., 2016). Body mass index (BMI) was calculated, with 23 or more kg/m^2^ defined as overweight or obesity. 

*Chronic conditions*

Chronic medical condition was assessed with the question, “Has a doctor/paramedic/nurse/midwife ever told you that you had…?” (“Hypertension, Diabetes or high blood sugar, Asthma, Other lung conditions, High cholesterol (total or LDL), Heart attack, coronary heart disease, angina or other heart problems, Stroke, Arthritis or rheumatism, Stomach or other digestive disease”) (Yes, No) (Strauss et al., 2016).

*Hypertension*

Based on averaged three consecutive measurements of systolic and diastolic blood pressure (Strauss et al., 2016), “hypertension was defined as SBP ≥140 mm Hg and/or DBP ≥90 mm Hg and/or current use of antihypertensive medication” (Chobanian et al., 2003).

The Centres for Epidemiologic Studies Depression Scale (CES-D: 10 items) was used to assess depressive symptoms, and scores 10 or more were classified as moderate to severe depressive symptoms (Andresen et al., 1994) (Cronbach alpha 0.67).

Self-reported health status was measured with the question, “In general, how is our health?” Response options were “1=Very healthy, 2, Somewhat healthy, 3=Somewhat unhealthy, and 4=Unhealthy” (Strauss et al., 2016). Those who responded somewhat unhealthy or unhealthy were defined as having poor self-rated health.

Functional disability was measured by five items of Activities of Daily Living (ADL) (Cronbach alpha 0.84) and six items of Instrumental Activities of Daily Living (IADL) (Cronbach alpha 0.91) (Katz et al., 1993; Lawton and Brody, 1969). Functional disability defined as having difficulty in one or more ADL/IADL items.

Fruit and vegetable consumption was assessed with questions on, “How many days in the past week did you eat, 1a) Green leafy vegetables? 1b) carrots? 2a) banana? 2b) papaya? 2c) mango?” (Strauss et al., 2016) Inadequate fruit consumptions was defined as “less than three days a week, and inadequate vegetable consumption less than daily”. 

Cognitive functioning was assessed with questions from the “Telephone Survey of Cognitive Status (TICS)” (Herzog and Wallace, 1997; Strauss et al., 2016). Total scores ranged from 0-34; scores 13 and lower were defined as low.

*Data analysis*

Descriptive statistics were calculated to describe the sample, and current and former tobacco use levels. Multinomial logistic regression was performed to estimate associations between all independent variables (sociodemographic and health related factors) and dependent variables of current and former tobacco use; with never tobacco use as reference category. Potential multi-collinearity between variables was assessed with variance inflation factors, none of which exceeded critical value. P < 0.05 was considered significant. Cross-section analysis weights were applied to make the study sample representative of the 2014 Indonesian population in the study provinces (Strauss et al., 2016). Both the 95% confidence intervals and P-values were adjusted considering the survey design of the study. All analyses were done with STATA software version 13.0 (Stata Corporation, College Station, TX, USA).

## Results

*Sample and tobacco use characteristics*

The total sample included 8001 older adults, 50 years and older (mean age 61.8 years, SD=9.8, age range of 50-110 years) in Indonesia. The proportion of women was 51.9%, 72.1% of the sample had no or elementary education, 42.4% described themselves as having medium economic status, 52.1% resided in urban areas and 58.1% lived in Java. Regarding health variables, 46.9% were overweight or obese, 8.1% high total cholesterol or low-density lipoprotein (LDL), 58.5% had hypertension, 2.9% had a stroke, 3.6% heart attack, angina or other heart problems, 46.9% overweight or obese, 11.8% arthritis, 6.3% had diabetes, 3.4% asthma, 2.3% other lung conditions and 17.0% depression symptoms. One in three of the participants (32.9%) rated their health as poor, 31.5% had a functional disability, 29.3% low cognitive functioning and 33.5 inadequate fruit and vegetable consumption. The overall prevalence of current tobacco use was 33.3% (62.2% in men and 6.5% in women) and former tobacco use was 9.8% (17.4% among men and 2.8% in women), of which 64.4% quit tobacco use when 50 years and older. Smoking tobacco (94.4%) was the most prevalent type of current tobacco use, while smokeless tobacco use was 5.6%. Among the different tobacco users, 39.6% of women and 1.3% of men used smokeless tobacco currently. The prevalence of smokeless tobacco use increased by age group, 2.0% in the age group 50-59 years, 3.6% 60-69 years, 13.5% 70-79 years and 26.6% in the age group 80 years and older (see Table 1).

**Table 1:** Sample Characteristics and Prevalence of Tobacco Use

Variable	Sample	Never tobacco use	Current tobacco user	Former tobacco user
	N (%)	n (%)	n (%)	n (%)
All	8,001	4,667 (56.9)	2,501 (33.3)	833 (9.8)
Age (years)				
50-59	4,027 (52.6)	2,411 (57.3)	1,321 (35.5)	295 (7.2)
60-69	2,229 (27.9)	1,301 (57.7)	682 (31.9)	246 (10.3)
70 and over	1,745 (19.5)	955 (54.7)	498 (29.2)	295 (16.0)
Gender				
Female	4,321 (51.9)	3,901 (90.7)	297 (6.5)	123 (2.8)
Male	3,680 (48.1)	766 (20.5)	2,204 (362.2)	710 (17.4)
Education				
None or elementary	5,636 (72.1)	3,360 (58.2)	1,768 (33.4)	508 (8.4)
High school or Higher education	2,295 (27.9)	1,269 (53.6)	711 (33.1)	315 (13.3)
Subjective economic background				
Poor	2,072 (31.0)	1,127 (53.8)	791 (39.2)	154 (7.0)
Medium	2,846 (42.4)	1,608 (54.4)	927 (35.2)	311 (10.5)
Rich	1,789 (26.7)	1,166 (63.8)	453 (27.4)	170 (8.7)
Residence				
Rural	3,543 (47.9)	1,918 (53.8)	1,288 (37.6)	337 (8.6)
Urban	4,458 (52.1)	2,749 (59.8)	1,213 (29.3)	496 (10.9)
Region				
Sumatra	1,661 (20.8)	881 (50.3)	568 (36.9)	212 (12.8)
Java	4,649 (58.1)	2,777 (58.0)	1,427 (32.9)	445 (9.2)
Major island groups	1,691 (21.1)	1,009 (58.2)	506 (31.3)	176 (10.5)
Health variables				
Overweight or obese	3,532 (46.9)	2,432 (56.9)	758 (33.6)	342 (9.5)
High total cholesterol or LDL	664 (8.1)	457 (66.5)	103 (17.3)	104 (16.2)
Hypertension	4,392 (58.5)	2,700 (60.6)	1,202 (28.7)	490 (10.7)
Stroke	244 (2.9)	144 (58.0)	38 (16.0)	62 (26.0)
Heart problems	299 (3.6)	188 (61.8)	37 (13.7)	74 (24.5)
Arthritis/rheumatism	1,038 (11.8)	677 (62.7)	256 (27.3)	106 (10.0)
Diabetes	506 (6.3)	324 (63.1)	103 (21.6)	79 (15.4)
Asthma	284 (3.4)	150 (50.7)	66 (26.1)	68 (23.2)
Other lung conditions	188 (2.3)	103 (54.1)	33 (17.9)	52 (27.9)
Cancer or malignant tumour	66 (0.8)	47 (71.4)	14 (21.4)	5 (7.1)
Stomach/digestive diseases	908 (11.5)	585 (62.6)	204 (24.4)	118 (13.0)
Depression symptoms	1,167 (17.0)	681 (55.9)	394 (36.7)	92 (7.4)
Poor self-rated health	2,852 (32.9)	1,696 (57.7)	784 (29.5)	372 (12.8)
Cognitive functioning (low)	1,630 (29.3)	920 (55.7)	552 (35.4)	158 (9.7)
Functional disability	2,642 (31.3)	1,499 (54.4)	762 (31.8)	381 (13.8)
Infrequent fruit and vegetable consumption	2,119 (33.5)	1,155 (53.0)	762 (38.2)	202 (8.9)

**Table 2:** Associations between Sociodemographic, Health Vvariables and Current and Former Tobacco Use

Variable	Current tobacco user ARRR (95% CI)1	Former tobacco user ARRR (95% CI)1
Age (years)		
50-59	1 (Reference)	1 (Reference)
60-69	0.85 (0.68, 1.05)	1.29 (0.98, 1.70)
70 and over	1.03 (0.72, 1.46)	2.92 (1.94, 4.39)***
Gender		
Female	1 (Reference)	1 (Reference)
Male	71.54 (54.50, 93.92)***	66.09 (44.71, 97.72)***
Education		
None or elementary	1 (Reference)	1 (Reference)
High school or Higher education	0.68 (0.55, 0.85)***	0.90 (0.68, 1.19)
Subjective economic background		
Poor	1 (Reference)	1 (Reference)
Medium	0.80 (0.64, 1.00)	1.25 (0.92, 1.71)
Rich	0.62 (0.48, 0.80)***	0.88 (0.62, 1.26)
Residence		
Rural	1 (Reference)	1 (Reference)
Urban	0.75 (0.61, 0.01)**	1.11 (0.85, 1.46)
Region		
Sumatra	1 (Reference)	1 (Reference)
Java	0.50 (0.39, 0.63)***	0.35 (0.26, 0.48)***
Major island groups	0.42 (0.32, 0.56)***	0.43 (0.30, 0.62)***
Health variables		
Overweight or obese	0.52 (0.43, 0.64)***	0.95 (0.72, 1.25)
High total cholesterol or LDL	0.97 (0.69, 1.37)	1.55 (1.04, 2.30)*
Hypertension	0.88 (0.73, 1.07)	1.13 (0.87, 1.46)
Stroke	0.33 (0.16, 0.67)**	0.97 (0.47, 1.98)
Heart problems	0.60 (0.35, 1.05)	1.89 (1.11. 3.23)*
Arthritis/rheumatism	0.98 (0.72, 1.33)	0.85 (0.57, 1.29)
Diabetes	0.73 (0.51, 1.06)	1.08 (0.69, 1.70)
Asthma	1.14 (0.63, 2.08)	2.24 (1.19, 4.23)*
Other lung conditions	0.38 (0.20, 0.71)**	1.34 (0.73, 2.44)
Cancer or malignant tumour	2.69 (1.12, 6.47)*	1.96 (0.64, 6.03)
Stomach/digestive diseases	1.10 (0.81, 1.51)	1.64 (1.11, 2.45)*
Depression symptoms	1.43 (1.08, 1.62)*	0.96 (0.66, 1.40)
Poor self-rated health	1.09 (0.87, 1.36)	1.10 (0.83, 1.46)
Cognitive functioning	1.00 (0.98, 1.03)	0.99 (0.96, 1.02)
Functional disability	1.32 (1.06, 1.65)*	1.62 (1.22, 2.14)***
Infrequent fruit and vegetable consumption	1.31 (1.06, 1.62)*	1.25 (0.95, 1.65)

ARRR, Adjusted Relative Risk Ratio; 1All variables in the Table are adjusted for; ***P<0.001; **P<0.01; *P<0.05

*Associations with current and former tobacco use*

In adjusted multinomial regression analysis, sociodemographic factors (being male, lower education, lower economic status, living in Java and rural residence) and health variables (cancer or malignant tumour, depression symptoms, functional disability and inadequate fruit and vegetable consumption) were associated with current tobacco use among older adults. In addition, being overweight or obese, having had a stroke, and other lung conditions were inversely associated with current tobacco use. Further, in adjusted analysis, sociodemographic factors (being 70 years and older, being male, living in Sumatra) and having chronic conditions (dyslipidaemia, Heart attack, coronary heart disease, angina or other heart problems, asthma, stomach or digestive diseases and functional disability) were associated with former tobacco use (see Table 2).

## Discussion

The study found a high prevalence of current tobacco use among older adults in Indonesia (33.3%; 62.2% in men and 6.5% in women), which seem to be lower than in the latest (2011) Global Adult Tobacco Survey in Indonesia (more than 38%) (WHO, 2012), but higher than in many other countries (He et al., 2012; Pang et al., 2016; Marinho et al., 2010). On the other hand the proportion of former tobacco use (9.8%; 17.4% among men and 2.8% in women) was low in this study, compared to studies in Ghana, Mexico, Russia and South Africa (He et al., 2012), but was similar to the low rates among older adults in China and India (He et al., 2012). Reasons for a possible decline of the prevalence of current tobacco use among older adults from 2011 to 2014/15 could be that a higher proportion of older adults quit tobacco use in the latest survey, which was 64.4% when they were 50 years and older, compared to a smoking quit rate of 51.5% within the past 10 years of persons 65 years and older in the 2011 survey (WHO, 2012). The study found some regional differences in the prevalence of current and former tobacco use, with higher rates in Sumatra compared to Java and major island groups. Reasons for this need further investigations. Intensified public health interventions are needed in targeting tobacco use in older adults in Indonesia.

The study found that current tobacco use did not significantly decline with increasing older age, which was also found in a study in South Africa (Van Heerden et al., 2009), while most studies seem to show a decline of current tobacco use with increasing age (Blazer and Wu, 2012; Kim et al., 2007). On the other hand, older age was in this study, in agreement with previous studies (Pang et al., 2016; Yawson et al., 2013) associated with former tobacco use. The strong male preponderance of current and former tobacco use is also in line with previous studies (He et al., 2012; Mini et al., 2014; Peltzer and Phaswana-Mafuya, 2012; Yawson et al., 2013), especially in low- and middle-income countries. These huge gender differences seem to reflect social norms in Indonesian or Asian culture being taboo for women to smoke (Mini et al., 2014; Pang et al., 2016). This finding is reinforced by the fact that a large proportion (39.6%) of older Indonesian women engaged in smokeless tobacco use. Although there was no significant difference in terms of age group and tobacco use, there was with ageing a significant increase in the use of smokeless tobacco, from 2.0% among 50-59 year-olds to 26.6% among individuals 80 years and older. Similar gender differences and increases of smokeless tobacco use with age have also been found in the 2011 Indonesia Global Adult Tobacco Use survey (WHO, 2012).

Lower socioeconomic and/or educational status was in this study associated with current tobacco use, as found in previous studies (Mini et al., 2014; Pang et al., 2016). However, contrary to a previous study in Ghana (Yawson et al., 2013), this study did not find an association between higher levels of education, increasing income levels and former tobacco use. Just like in several other middle-income countries (China, Ghana, and India) (He et al., 2012; Mini et al., 2014), this study found an association between rural residence and current tobacco use. The prevalence of former tobacco users was in this study higher in urban than rural areas, as found in a study in Ghana (Yawson et al., 2013), but this was statistically not significant.

Several chronic conditions (cancer or malignant tumour, depressive symptoms, functional disability, dyslipidaemia, heart problems, asthma, stomach and digestive diseases) were in this study positively associated with current and/or former tobacco use. This has been partially also found in previous studies (Burns et al., 2017; Pang et al., 2016; Sumartono et al., 2011). Former tobacco users were significantly more likely to have five chronic conditions measured in this study, while current smokers had only three chronic conditions compared to never tobacco users. A possible reason for this is that tobacco users tend to quit after receiving a diagnosis of a chronic condition, likely to be related to tobacco use (Pang et al., 2015).

Overweight or obesity was in this study negatively associated with current tobacco use, as also found in previous studies (e.g. Peltzer and Phaswana-Mafuya, 2012; Pengpid and Peltzer, 2017). Some research refers to nicotine intake as promoting satiety so that overweight development may be reduced (John et al., 2006).

Having had a stroke and having other lung conditions (other than asthma) were in this study negatively associated with current tobacco use. It is possible that due to stroke and/or having lung conditions tobacco users quit tobacco use. This may be confirmed by the positive (but not significant) association between having other lung conditions and former tobacco use. Tobacco use may exacerbate existing chronic conditions in the increasingly ageing older population in Indonesia calling for a critical need for tobacco cessation programming (Yawson et al., 2013).

In agreement with several previous studies (Ko et al., 2011; Peltzer and Phaswana-Mafuya 2012), this study found an association between insufficient fruits and vegetable consumption current tobacco use. It is possible that unhealthy behaviours such as inadequate fruit and vegetable consumption clusters with other risk behaviours such as current tobacco use. Unlike some previous studies (Bell et al., 2009; Mons et al., 2013), this study did not find an association between poor self-rated health, poor cognitive functioning and tobacco use. 

*Limitations of the study*

The self-report of tobacco use has its limitations, especially among women and older adults. It is possible that individuals who reported not being a tobacco user misclassified their tobacco use status (Al-Houqani et al., 2018; Moradzadeh et al., 2018). Some indicators such as alcohol use and other drug use, formerly found associated with tobacco use (Blazer and Wu, 2012; Dani and Harris, 2005) were not assessed in this study and should be included in future investigations. In addition, this study used a cross-sectional design and therefore, we cannot ascribe causality to any of the associated factors in the study. 

In conclusion, this study reports high rates of current and low rates of former tobacco use among older adults (50 years and more) in Indonesia that puts them at increased risk of morbidity and mortality. Tobacco use among older adults is often under-recognized needing multilevel (health care and educational) interventions. 

## Conflict of interest statement

None declared.
